# Programmed Death Ligand-1 Expression in Gastric Cancer Cases in Zambia

**DOI:** 10.53974/unza.jabs.9.2.1492

**Published:** 2025-06-04

**Authors:** Husna Munshi, Chibamba Mumba, Mupeta Songwe, Violet Kayamba

**Affiliations:** 1 Department of Pathology and Microbiology, University of Zambia School of Medicine, Lusaka, Zambia.; 2 Tropical Gastroenterology & Nutrition group, Lusaka, Zambia; 3 Department of Pathology and Microbiology, University Teaching Hospital, Lusaka, Zambia.; 4 Department of Internal Medicine, University of Zambia School of Medicine, Lusaka Zambia

**Keywords:** Gastric Cancer, Adenocarcinoma, Immunohistochemistry, Programmed Death Ligand-1, Immunotherapy, Zambia

## Abstract

Gastric cancer is a highly fatal disease in Zambia due to delayed diagnosis, aggressiveness of the disease and ineffective treatment. Programmed death ligand-1 is a key biomarker of gastric cancer, linked to immune evasion and response to anti-Programmed death ligand-1 therapies. This study aimed to evaluate the expression of in gastric cancer cases and its association with various clinicopathological prognostic factors in Zambia.

This pilot study utilized archived formalin-fixed, paraffin-embedded tissue blocks from patients diagnosed with gastric cancer at the University Teaching Hospitals in Lusaka, Zambia. A total of 41 gastric cancer samples were examined, with 65.9% female (27) and 34.1% male (14), and a median age of 63 years (interquatile range: 56–76). The slides were stained with Haematoxylin and Eosin, followed by immunohistochemical analysis to assess Programmed Death Ligand-1 expression, which was evaluated using a combined positive scoring system. Statistical analysis was conducted using STATA to evaluate the data version 15.

Six patients (14.6%) exhibited positive expression of Programmed Death Ligand-1, with a combined positive score of 1 or higher. However, our analysis did not reveal any significant associations between PD-L1 expression and any of the clinicopathological variables assessed. We found that a small proportion (14.6 %) of GCs in our population expresses Programmed Death Ligand-1, a potentially therapeutically actionable patient group.

## 1.0 Introduction

Gastric cancer (GC) ranks fifth globally in incidence and mortality (1–3). In Zambia, the situation is particularly dire, with a one-year survival rate of less than 15%, highlighting the severity of the problem and the need for improved healthcare interventions (4).

Many gastrointestinal tumours, exhibit aberrant programmed death ligand 1 (PD-L1) expression. (5,6). PD-L1 is an essential immune checkpoint transmembrane protein that binds to its receptor, programmed death 1 (PD-1) and is physiologically involved in immune modulation (7,8). The binding of PD-L1 to PD-1 on T cells disrupts signalling pathways, leading to reduced immune surveillance, T-cell exhaustion, and apoptosis, which allows tumour cells to evade immune detection (9–11). Recent research has uncovered promising results, such as enhanced overall survival and/or progression-free survival, among patients with advanced GC expressing the PD-L1 marker who received treatment with anti-PD-1/ PD-L1 inhibitors, a class of immune checkpoint inhibitors (ICIs) (12,13). The introduction of ICIs has revolutionised advanced cancer therapy, establishing them as a cornerstone of immunotherapy across various tumour types including GC. In many developed countries, ICIs have become a standard treatment for advanced cancers, including GC(9,14).

In Zambia, treatment options for GC are currently limited to surgery, chemotherapy, and radiotherapy, as molecular sub-typing is not routinely available and treatment decisions rely solely on basic histologic tumour morphology. Immunotherapy targeting PD-L1 is not yet practiced in Zambia, and the prevalence of PD-L1 expression in GC patients remains unknown, resulting in a lack of evidence to support the use of anti-PD-1/PD-L1 immunotherapy. This limited molecular data and restricted treatment options likely contribute to poor patient outcomes. Therefore, this study analysed archival gastric biopsies from Zambian adults to assess PD-L1 expression in GC and its potential links to clinicopathological features, aiming to guide future research and broaden treatment options in Zambia and clinicopathological features associated with disease prognosis.

## 2.0 Materials and Methods

### 2.1 Study Population

This was a pilot study determining the expression of PD-L1 from archival GC biopsies. The patient cohort has been previously described in a case-control study (15). Briefly, gastric biopsy samples were obtained at the time of endoscopy from patients that presented with dyspepsia at the University Teaching Hospitals (UTHs), Lusaka, Zambia, from 2016 to 2023, and were stored as formalin fixed paraffin embedded tissue blocks. In the study, cases were defined as those that were diagnosed with GC during endoscopy and those without as controls.

A total of 138 GC cases were identified in the parent study database of which 84 formalin fixed paraffin embedded (FFPE) tissue blocks were retrieved from the archive as depicted in Figure 1.0. Specimens lacking sufficient tumour tissue, exhibiting tissue distortion, or possessing incomplete clinicopathological information were excluded from analysis. Consequently, 41 cases met the criteria for the final PD-L1 immunohistochemical (IHC) analysis. Clinicopathological data (including age, gender, occupation, tumour site, *H.pylori* infection status, degree of differentiation and Lauren classification) were annotated for eligible cases from the parent study and de-identified.

The study was approved by the University of Zambia Biomedical Ethics Committee and the National Health Research Authority, reference number: 000-03-16. Written informed consent was obtained from all the subjects involved in the study.

### 2.2 Immunohistochemistry (IHC)

Tumour specimens were processed by cutting 4-μm-thick sections from paraffin-embedded blocks, followed by de-parafinisation and rehydration. Antigen retrieval was performed using a high pH citrate buffer in pressure cooker at 120°C for 40 minutes. After cooling sections were treated with peroxidase blocking solution before being incubated overnight with primary antibody (Monoclonal Mouse Anti-Human PD-L1, clone 22C3, Dako, Agilent Technologies, Santa Clara, USA) at a 1:100 dilution. Next, the slides were incubated with a Mouse Linker (Dako, USA) followed by Horse Radish Peroxidase (HRP) and stained with 3–3’-diaminobenzidine (DAB) and counterstained using hematoxylin. The slides were rinsed with wash buffer (Envision Flex Wash Solution 10X Concentrate, Dako, USA) after each step. Appropriate quality controls were applied during the staining of each batch (Placenta and tissues from penile squamous cell carcinoma with known positive expression of PD-L1). The procedure adhered to the manufacturer’s guidelines, with optimisations implemented accordingly.

### 2.3 IHC analysis and scoring

Scoring was done according to the Agilent/Dako 22C3 pharmDx assay for gastric cancer guidelines (Agilent Technologies, Santa Clara, USA) and the combined positive score (CPS) was quantified as described by the manufacturer. The PD-L1 CPS is currently recognized as a screening tool that is easy to apply clinically (16). It is calculated by dividing the number of PD-L1 stained cells (tumour cells and immune cells) by the total number of viable tumour cells and by multiplying the value by 100. Negative expression of PD-L1 was defined as CPS < 1, and positive PD-L1 expression was defined as a CPS ≥ 1. Positive PD-L1 expression was further graded into three groups: low expression (CPS ≥1 to <10), moderate expression (CPS ≥10 to <15), and strong expression (CPS ≥15).

Analysis of the IHC stained slides was carried out by two independent certified pathologists who were blinded to the clinical and pathological details of the patients’ samples. The pathologists re-examined any discordant interpretations until consensus was achieved.

### 2.4 Statistical analysis

Collected data were entered into an excel spread sheet and later exported to STATA version 15 (College Station, TX, USA). All continuous variables were first checked for normality using the Shapiro-Wilk test. Means and standard deviations were used to summarise normally distributed continuous variables while medians and inter-quartile ranges were used for skewed variables. Categorical variables were presented in percentages. Associations were assessed using Fisher’s exact test and Kruskal-Wallis tests. Odds ratios with 95% confidence intervals were derived with PD-L1 positivity as study outcome and clinic-pathologic characteristics as the exposure variable. All *p* values less than 0.05 were considered statistically significant.

## 3.0 RESULTS

### 3.1 Expression of PD-L1 in Gastric Cancer Patients and Its Relation to Clinicopathological Variables

Out of the 41 gastric tissues analyzed, 27 (65.9%) were from females, and 14 (34.1%) were from males (Table 1). The median age at diagnosis was 63 years (IQR 56–76). Using a cut-off value of CPS ≥1, 6 (14.6%) patients were identified as PD-L1 positive, while 35 (85.4%) were negative (CPS <1).

Table 1 summarises the characteristics of patients according to tumour PD-L1 expression. Most patients that exhibited positive PD-L1 expression were females (83.3%); patients who were above the age of 45 years; underweight patients (66.6%) and those that tested positive for *H.pylori* infection (66.6%). The poorly differentiated intestinal type was the most common tumour histology observed, regardless of PD-L1 expression status. None of the factors showed statistical significance, as all p-values exceeded 0.05.

### 3.2 PD-L1 Expression Ranges and Immunohistochemical Staining in Gastric Cancer Patients

Among the PD-L1 positive samples, 50% (3/6) showed strong expression (CPS ≥15), 16.7% (1/6) showed low expression (CPS ≥1 to <10), and the remaining samples exhibited moderate expression (CPS ≥10 to <15) as shown in Figure 2.0. The IHC staining pattern and intensity of the PD-L1 marker in GC tissues is depicted in the images presented in Figure 2.0.

## 4.0 Discussion

This study set out to investigate the PD-L1 expression in GC within the Zambian population and explore possible associations between PD-L1 and various demographic and clinicopathological variables in a small well characterized cohort of GC patients. We observed on 14% patients were positive for PD-L1 and none of the clinic-pathologic factors were statistically associated with PD-L1 expression in the GC tumour samples. Our positive PD-L1 expression was in agreement with a report from Morocco (15.8%) (17) using a cut-off value of ≥1. Meanwhile, other studies described higher frequencies of PD-L1 positivity ranging from 43.2% in Egypt (18), and up to 71.7% in Japan, South Korea and Jordan (19–21). Such discrepancies in PD-L1 expression could be ascribed to variations in the patient population, interpretation of staining patterns, scoring methods, adopted cut-off values, and the diverse monoclonal antibodies employed by different researchers. Yeong *et al* conducted a comparative study that revealed the percentage of PD-L1-positive samples at clinically relevant CPS cut-offs (≥ 1, ≥ 5, and ≥ 10) for the 28–8 assay, and found that it was approximately twice as high as that observed with the 22C3 assay (14). Additionally, GC is molecularly heterogeneous, necessitating consideration of this factor in assessing predictive biomarkers such as PD-L1, whose expression may differ among various molecular subgroups of GC (10). Understanding specific GC subtypes is crucial due to molecular diversity, as data suggests that patients with Epstein-Barr virus-associated (EBV+) and microsatellite instability-high (MSI-H) tumours, which are linked to higher PD-L1 expression, may respond to anti-PD-1/PD-L1 therapy (22,23) This potential responsiveness may be linked to the high mutational burden and neo-antigen generation associated with MSI-high tumours (24). In another study we conducted in Zambia, we found a high occurrence of MLH1 loss, indicating potential microsatellite instability, which has been previous show to have higher PD-L1 expression and responsiveness to anti-PD-1/PD-L1 therapy in gastric cancer.(25).

High CP-scores have been associated with a higher chance of having positive clinical outcomes from PD-1/PD-L1 inhibitors (14,26). The notably strong expression of PD-L1 (CPS ≥15) in majority (50.0%) of positive tumours observed in our study may suggest a greater potential for positive benefits from ICIs in this specific subgroup of patients.

**Figure 2:**
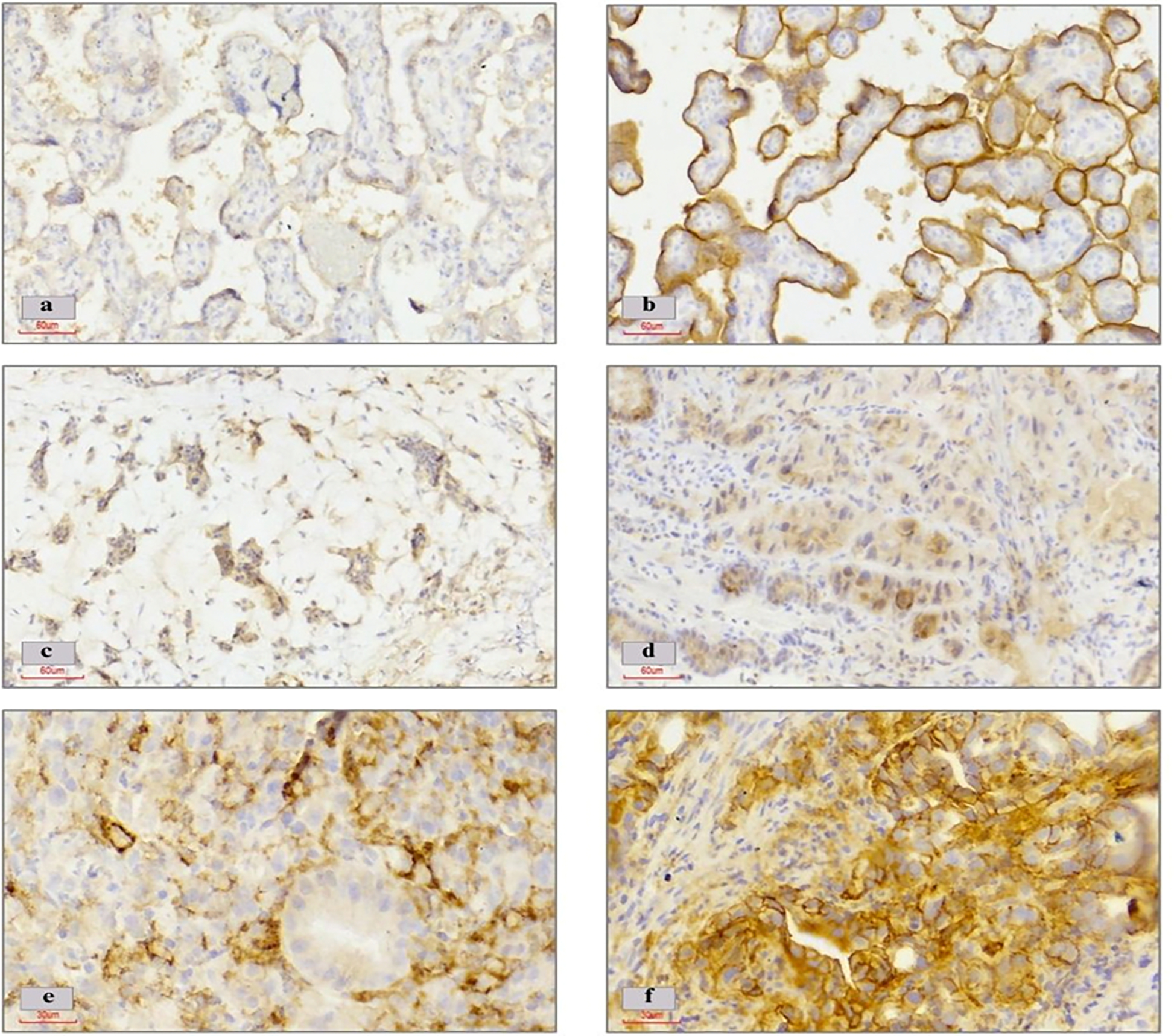
Immunohistochemical staining for programmed death ligand-1 (PD-L1) in gastric cancer tissues. Figure 2(a) and 2(b) depict negative and positive tissue controls for PD-L1 expression in placental tissue, respectively (magnification ×200). GC samples were categorized into three groups based on PD-L1 expression: Figure 2(c) shows no expression (CPS<1) - islands of cells with no staining with PD-L1 antibody (magnification ×200). Figure 2(d) displays a low expression (CPS ≥1 to <10) - few cells with membrane staining using PD-L1 antibody (magnification ×200). Figures 2(e) and 2(f) demonstrate moderate (CPS ≥10 to <15) and strong (CPS ≥15) expression, respectively, illustrating several cells with membranous staining with PD-L1 antibody (magnification ×400).

Lack of PD-L1 expression in the tumour on the other hand, as illustrated in figure 2(c), has been associated with a higher chance of survival in patients compared to those with positive expression, who are consistently linked to an unfavorable prognosis (27–29). In a univariate analysis by Zhang *et al*, individuals with negative expression showed a significantly longer survival rate, with a 32.4% higher rate compared to those expressing the marker positively (83.1% versus 50.7%) (7). The significant survival rate disparity between the two groups highlights the need for interventions to improve outcomes for the group with poorer survival. It is also crucial to recognize that the presence of PD-L1 expression does not universally predict a favourable response to immune checkpoint inhibitors, as some PD-L1-negative tumours may nonetheless derive benefit from such therapies (1). However, due to the use of archival samples and the absence of patient follow-up, survival data analysis was not feasible in our study.(1). However, due to working with archival samples and lack of patient follow-up, our study did not include survival data analysis.

The present study also explored the relationship between PD-L1 expression and various clinicopathological factors, including sex, age, occupation, education level, *Helicobacter pylori* infection status, tumour location, and histological subtype. No statistically significant correlations were observed for any of these variables. These findings align with previous reports by Sughayer *et al.* and Yeong *et al.,* who similarly found no significant associations between PD-L1 expression and clinicopathological parameters(14,19). However, in contrast, Attia *et al.* identified a statistically significant correlation between PD-L1 expression and increasing patient age, suggesting that age may influence PD-L1 expression in some cohorts (18). This divergence highlights the complexity of PD-L1’s role in tumor biology and its variable association with patient demographics, likely due to differences in sample size, study design, and tumour characteristics.

Despite these insights, the main limitation of our study stems from its relatively small sample size, consisting of only 41 GC patients from a single institution. Furthermore, our analysis was based on biopsy samples rather than whole ressected tissues, which may have restricted the comprehensive assessment of PD-L1 expression across the tumour. The limited biopsy material might not fully capture the heterogeneity of the biomarker, potentially leading to an incomplete representation when compared to ressected tissue samples. In addition, critical information regarding tumour stage—a key determinant of prognosis and treatment response—was unavailable, limiting our ability to contextualize PD-L1 expression within the broader clinical picture.

## Conclusion

In conclusion, this pilot study found that 14.6% of GC patients in Lusaka express PD-L1, with no statistically significant link to clinicopathological factors. Comprehensive PD-L1 evaluation may aid in selecting patients for targeted therapies, and these results provide a foundation for further studies to improve diagnostic methods.

## Figures and Tables

**Figure 1:**
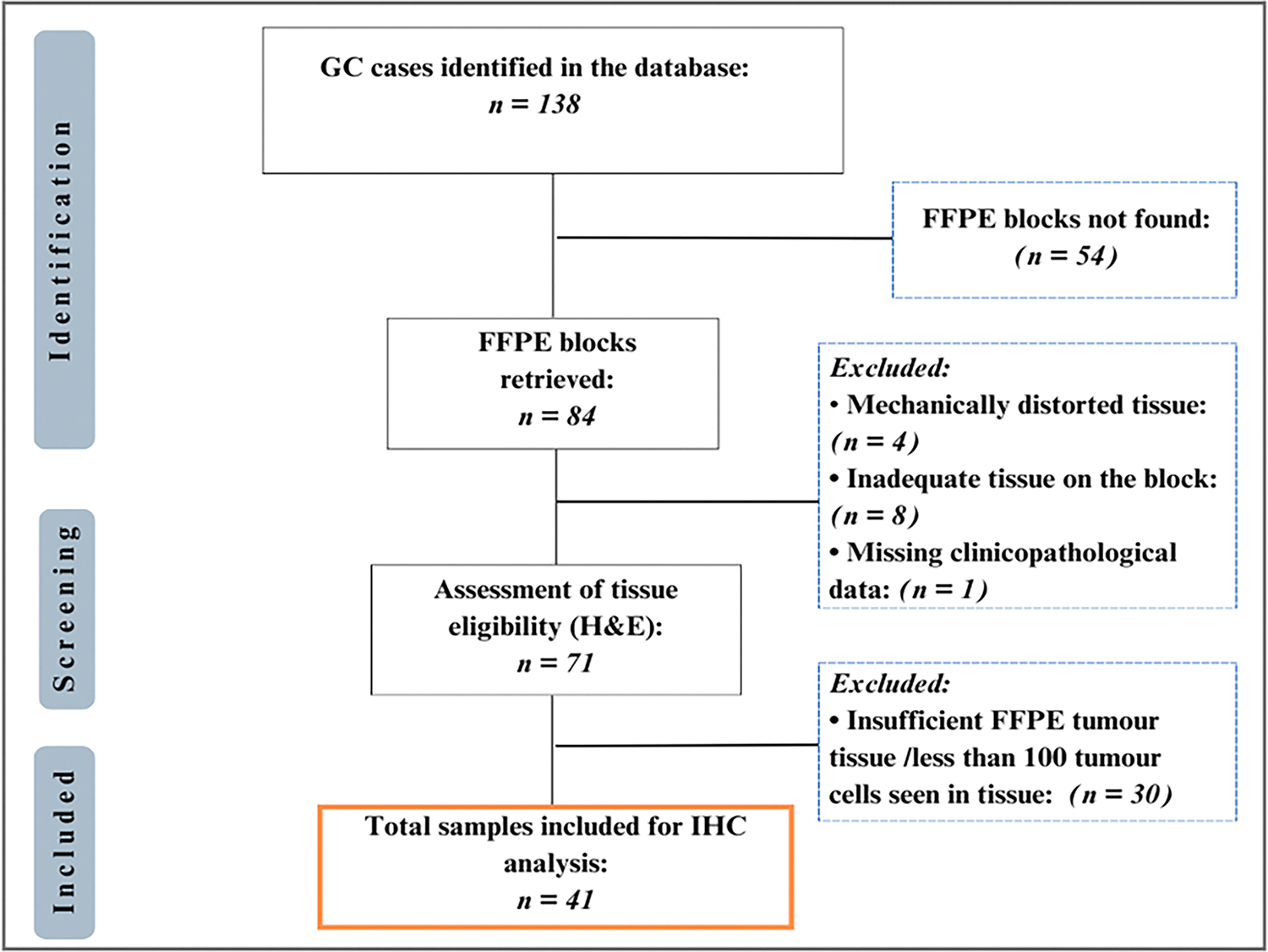
Flowchart illustrating sample selection for programmed death ligand-1 Immunohistochemical analysis.

## APPENDIX B: TABLES

**Table 1:** Basic characteris<cs of the samples included in the analysis

Variable	n=41, N (%)	PD-L1 expression		OR; 95% CI	*P* value
		Positive (CPS ≥1), n=6 N (%)	Negative (CPS<1), n=35 N (%)		

Sex					
Male	14 (34.1)	1 (16.7)	13 (37.1)	2.95; 0.28 – 150	0.69*
Female	27 (65.9)	5 (83.3)	22 (62.9)		

Age in years,					
<45	63 (56 – 76)	0 (0.0)	5 (14.3)	0; 0 – 4.47	1.00*
≥45		6 (100.0)	30 (85.7)		

Education level				-	0.67
None	6 (14.6)	1 (16.7)	5 (14.3)		
Primary	19 (46.4)	3 (50.0)	16 (45.7)		
Secondary	10 (24.4)	2 (33.3)	8 (22.8)		
Tertiary	5 (12.2)	0 (0.0)	5 (14.3)		
Missing data	1 (2.4)	0 (0.0)	1 (2.9)		

Tumour site				-	1.00
GEJ/cardia	2 (4.9)	0 (0.0)	2 (5.7)		
Fundus	6 (14.6)	1 (16.7)	5 (14.3)		
Body	18 (43.9)	3 (50.0)	15 (42.9)		
Antrum	15 (36.6)	2 (33.3)	13 (37.1)		

Histology (Lauren classification)					
Intestinal type	24 (58.5)	4 (66.7)	20 (57.1)	-	0.56
Diffuse type	13 (31.7)	2 (33.3)	11 (31.4)		
Mixed type	4 (9.8)	0 (0.0)	4 (11.4)		

Histology (Tumour differentiation)					
Poorly differentiated	22 (53.7)	3 (50.0)	19 (54.3)	-	
Moderately differentiated	11 (26.8)	2 (33.3)	9 (25.7)		0.95
Well differentiated	7 (17.1)	1 (16.7)	6 (17.1)		
Undifferentiated	1 (2.4)	0 (0.0)	1 (2.9)		

*H. pylori* infection				1.5; 0.12 – 82.0	1.00*
Positive	28 (68.3)	4 (66.6)	24 (68.6)		
Negative	10 (24.4)	1 (16.7)	9 (25.7)		
Missing data	3 (7.3)	1 (16.7)	2 (5.7)		

Computed using *Fisher’s exact test and Kruskal-Wallis rank test

CI, Confidence Interval; CPS, combined positive score; IQR, Interquatile Range; OR, Odds ratio; PD-L1, programmed death ligand 1.
